# Contractility–Afterload Mismatch in Patients With Protein-Losing Enteropathy After the Fontan Operation

**DOI:** 10.1007/s00246-014-0920-8

**Published:** 2014-05-15

**Authors:** Hideto Ozawa, Takayoshi Ueno, Shigemitsu Iwai, Hiroaki Kawata, Kyouichi Nishigaki, Hidefumi Kishimoto, Yoshiki Sawa

**Affiliations:** 1 Department of Cardiovascular Surgery, Osaka University Graduate School of Medicine, 2-2 Yamada-oka, Suita, Osaka 565-0871 Japan; 2 Department of Cardiovascular Surgery, Osaka Medical Center and Research Institute for Maternal and Child Health, Izumi, Osaka Japan; 3 Department of Pediatric Cardiovascular Surgery, Osaka General Medical Center, Osaka, Osaka Japan

**Keywords:** Fontan, Protein-losing enteropathy, Contractility–afterload mismatch

## Abstract

This study aimed to clarify the relationship between onset of protein-losing enteropathy (PLE) and Fontan circulation, with special reference to the development of contractility–afterload mismatch. The PLE group comprised 9 patients who experienced PLE after undergoing the Fontan operation, and the control group consisted of 32 patients had did not experienced PLE more than 10 years after the Fontan operation. The study compared the pre- and postoperative values of arterial elastance (Ea), end-systolic elastance (Ees), and contractility–afterload mismatch (Ea/Ees). Furthermore, the variations in the values were examined during the preoperative, postoperative, and midterm postoperative periods in seven PLE patients who underwent cardiac catheterization at the onset of PLE and during the pre- and postintervention periods in three PLE patients who underwent surgical intervention to improve the Fontan circulation after the onset of PLE. Comparison of the values obtained before and after Fontan operations showed that the Ea values increased significantly in the PLE group. However, the pre- and postoperative Ees values did not differ in the two groups. During the postoperative period, Ea/Ees increased significantly, and the Ea and Ea/Ees values increased continuously until the onset of PLE in the PLE group. In the patients who underwent surgical intervention to improve the Fontan circulation after the onset of PLE, the Ea/Ees decreased significantly, and the serum albumin levels improved after the intervention. Contractility–afterload mismatch, mainly caused by the increase in the afterload of the systemic ventricle, may have an important role in the development of PLE after the Fontan operation.

Recently, early survival after the Fontan operation for a functional single ventricle has improved. However, long-term complications remain a problem [4, 11]. One such complication is protein-losing enteropathy (PLE), observed in 3–15 % of patients after the Fontan operation [2, 5]. The pathophysiologic mechanism of this disorder is poorly understood, but some reports have indicated that cardiac output was diminished after the Fontan operation and was even poorer in patients who experienced PLE after the operation [6].

Moreover, Szabo et al. [9] reported that Fontan circulation had profound effects on ventriculoarterial mechanoenergetics in terms of contractility–afterload mismatch and reduced mechanical efficacy. Contractility–afterload mismatch [increased arterial elastance (Ea)/end-systolic elastance (Ees)] was considered because of increased impedance (Ea) and decreased myocardial contractility (Ees) after the Fontan operation. We assumed that contractility–afterload mismatch caused PLE after the low cardiac output of the Fontan circulation. However, the degree of contractility–afterload mismatch in PLE patients compared with other patients who have the Fontan circulation has not been fully elucidated to date.

This study aimed to clarify the relationship between PLE onset and Fontan circulation, with special reference to the development of contractility–afterload mismatch.

## Patients and Methods

This study was a retrospective multicenter investigation. Between 1990 and 2011, 197 patients underwent the Fontan operation at three institutions. From these patients, we selected 9 who experienced PLE after the Fontan operation as the PLE group and 32 who had not experienced PLE more than 10 years after the Fontan operation as the control group. Informed consents were obtained from all parents of the patients.

The patient characteristics of both groups are summarized in Table 1. The two groups did not differ significantly in terms of anatomic diagnosis, age at operation, type of Fontan circulation, or percentage of the staged total cavopulmonary connection (TCPC). All the patients had undergone TCPC without fenestration. In the PLE group, PLE had developed a median of 4 years and 8 months (range 3 months–10 years) after the Fontan operation.

**Table 1:** Patients’ characteristics^a^

	PLE group	Control group
Total no. of patients	9	32
Diagnosis SV(RV/LV)	3(2/1)	12
DORV	1	8
MA-VSD	1	1
PAIVS	2	4
Ebstein	2	3
TGA		4
Age at Fontan operation	4 years 8 months	4 years 4 months
Fontan type		
Lateral tunnel TCPC	2	5
Extra cardiac TCPC	7	27
Staged TCPC: *n* (%)	7 (77)	26 (81)

There was no significant difference between the PLE and control groups

*PLE* protein-losing enteropathy, *SV* single ventricle, *RV* right ventricle, *LV* left ventricle, *DORV* double-outlet right ventricle, *MA-VSD* mitral atresia with ventricular septum defect, *PAIVS* pulmonary atresia with intact ventricular septum, *TGA* transposition of great arteries, *TCPC* total cavopulmonary connection

The diagnosis of PLE was based on a serum albumin level lower than 3.0 g/dL, a serum total protein level lower than 5.0 g/dL, and an elevated stool α-1 antitrypsin clearance or evidence of an abnormal enteric protein loss on technetium-99m (99mTc)-labeled human serum albumin scintigraphy. All the patients underwent cardiac catheterization before the Fontan operation and 1 year afterward. The ventricular volumes were calculated with area length methods by Kada-View software (Photron Medical Imaging Inc., Tokyo). The ventricular volume was normalized with the body surface area.

We compared the pre- and postoperative ventricular and arterial properties between the two groups using Ea and Ees, indices of afterload and myocardial contractility. The Ea/Ees ratio (ventriculoarterial coupling), an index of contractility**–**afterload mismatch, also was calculated. The approximations of Ea and Ees devised by Tanoue et al. [10] were as follows:

1 $${\text{Ea }} = \, \left( {\text{maximum ventricular pressure}} \right)/\left( {\left[ {\text{maximum ventricular volume}} \right] - \left[ {\text{minimum ventricular volume}} \right]} \right)$$

2 $${\text{Ees }} = \, \left( {\text{mean arterial pressure}} \right)/\left( {\text{minimum ventricular volume}} \right).$$

Both Ea and Ees were calculated using the volume and pressure data obtained during cardiac catheterization.

First, we examined variations in the Ea, Ees, and Ea/Ees values before the Fontan operation and then 1 year afterward. Second, we examined the variation in the Ea, Ees, and Ea/Ees values during the preoperative period, the 1-year postoperative period, and at the time of PLE onset. Because PLE occurred a median of 4 years and 8 months after the Fontan operation, we compared the values at the time of PLE onset with those of eight patients in the control group who had undergone cardiac catheterization 5 years after the operation. Finally, we also examined the variations in the Ea, Ees, and Ea/Ees values before and after the surgical interventions to improve the Fontan circulation after the onset of PLE. The patients’ numbers are summarized in Table 2.

**Table 2:** Number of patients included in the study

No. of patients	Before and 1 year after Fontan operation	Before, 1 year after, and long-term* after Fontan operation	Before and after intervention for PLE
PLE group	9	7	3
Control group	32	10	

Variance of the hemodynamic parameters before and 1 year after the Fontan operation were compared with 9 PLE patients and 32 control patients; the same parameters before, 1 year after the Fontan operation, and at midterm were compared with 7 PLE patients and 10 control patients

*PLE* protein-losing enteropathy

*At the time of PLE onset in PLE patients. We compared the values at the time of PLE onset with those of ten patients in the control group who had undergone cardiac catheterization 5 years after the operation

### Statistical Analysis

Continuous variables are presented as means ± standard deviations (SD’s) and categorical variables as frequencies. Continuous and categorical variables (hemodynamic parameters before and after the Fontan operation and complications after the Fontan operation) between the two groups were compared using the Mann-Whitney *U* test and Fisher’s exact test, respectively.

Changes in cardiac performance parameters, such as EDVI, CVP, CI, PAI, EDP, Ea, Ees, Ea/Ees, over time were compared between the two groups by repeated measures analysis of variance with the main effects of group and time and the interaction effect between them. All *p* values are two-sided, and values of *p* lower than 0.05 are considered to indicate statistical significance. Statistical analyses were performed using JMP 7.0 (SAS Institute, Cary, NC, USA) and SPSS version 17.0 (IBM Inc., Chicago, IL, USA).

## Results

No deaths were encountered during the study period. Two patients experienced PLE within 1 year after the operation. All the patients in the PLE group took diuretics, vasodilators, and steroids. Three patients underwent surgical intervention to improve the Fontan circulation after the diagnosis of PLE. One patient underwent takedown of the Glenn circulation because of the high central venous pressure (18 mmHg), and one patient underwent diaphragm plication for phrenic nerve paralysis. The latter patient underwent residual antegrade pulmonary flow occlusion.

Of the complications after the Fontan operation, systemic ventricle outflow tract stenosis (SVOTS) and sick sinus syndrome (SSS) were significantly more frequent in the PLE group. Although no significant difference was observed, phrenic nerve palsy also was more frequent in the PLE group, as indicated in Table 3. The diagnosis of SVOTS determined when the catheterization systolic pressure gradient between the systemic ventricle and the ascending aorta was higher than 10 mmHg.

**Table 3:** Complications after the Fontan operation

	PLE	Control	*p* Value
SVOTS	4/9	2/32	0.02
Phrenic nerve palsy	3/9	2/32	0.07
	4/9	3/32	0.03

The ratios of SVOTS, phrenic nerve palsy, and SSS were significantly higher in the PLE group than in the control group

*PLE* protein-losing enteropathy, *SVOTS* systemic ventricle outflow tract stenosis, *SSS* sick sinus syndrome

**Fig. 1:**
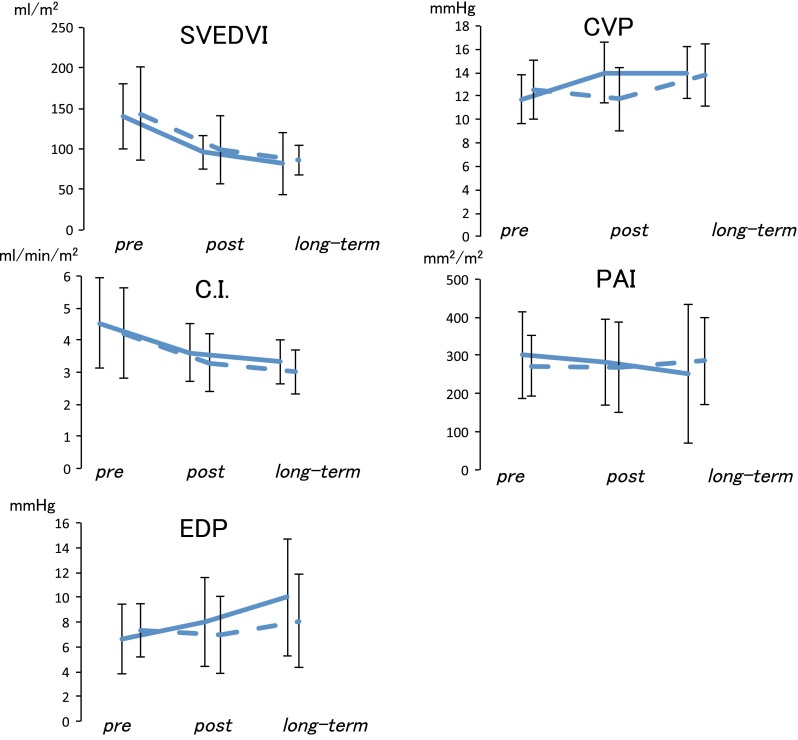
Hemodynamic parameters during follow-up evaluation. EDVI and CI decreased after the Fontan operation in both groups. However, the changes in EDVI and CI before and after the operation in the PLE and control groups did not differ significantly. On the other hand, EDP did not show a remarkable change in either group. *EDVI* end-diastolic volume index, *CI* cardiac index (mL/min/m^2^), *PLE* protein-losing enteropathy, *EDP* end-diastoic pressure (mmHg), *SVEDVI* systemic ventricle end-diastolic volume index (mL/m^2^), *CVP* central venous pressure (mmHg), *TPG* transpulmonary pressure gradient (mmHg), *BSA* body surface area, *PAI* pulmonary artery index (mm^2^/BSA)

The cardiac performance parameters in both groups before and after the Fontan operation are presented in Fig. 1. The systemic ventricular end-diastolic volume index (EDVI) and the cardiac index (CI) decreased after the Fontan operation in both groups. However, the changes in the EDVI and CI before and after the operation in the PLE and control groups did not differ significantly. On the other hand, the end-diastolic pressure did not show a remarkable change in either group.

**Fig. 2:**
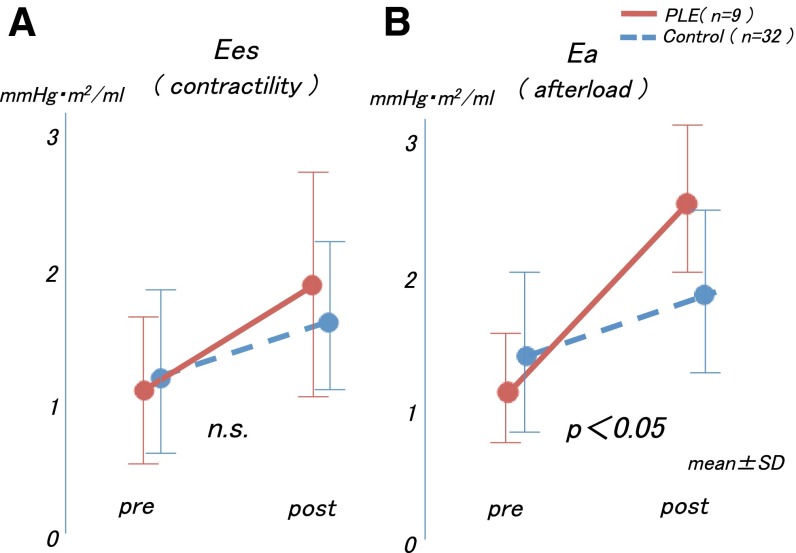
**a** The Ees values before and after the Fontan operations did not differ significantly between the two groups. **b** The increment of the Ea value was larger in the PLE group than in the control group. *Ees* end-systolic elastance, *Ea* arterial elastance, *PLE* protein-losing enteropathy

**Fig. 3:**
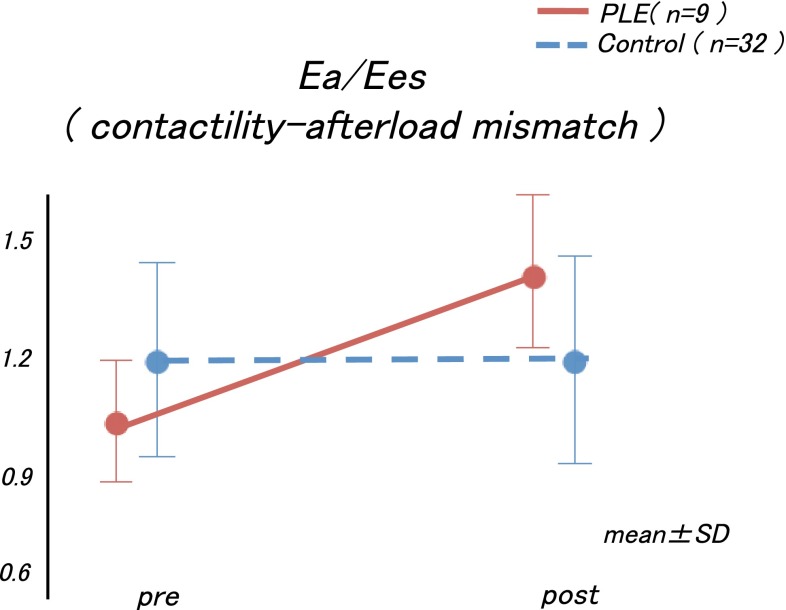
The increments in the Ea and Ea/Ees values were significantly higher in the PLE group than in the control group. *Ea* arterial elastance, *Ees* end-systolic elastance, *PLE* protein-losing enteropathy

Regarding the time course in the Ea, Ees, and Ea/Ees values, we observed that the Ees values before and after the Fontan operations did not differ significantly between the two groups (interaction effect, *p* = 0.97; group effect, *p* = 0.14; Fig. 2a). However, the time course in the Ea values differed significantly between the two groups (interaction effect, *p* = 0.001), and the increment of the Ea value was larger in the PLE group than in the control group (Fig. 2b). These changes caused an increase in the Ea/Ees values only in the PLE group after the Fontan operation (interaction effect, *p* = 0.03; Fig. 3).

**Fig. 4:**
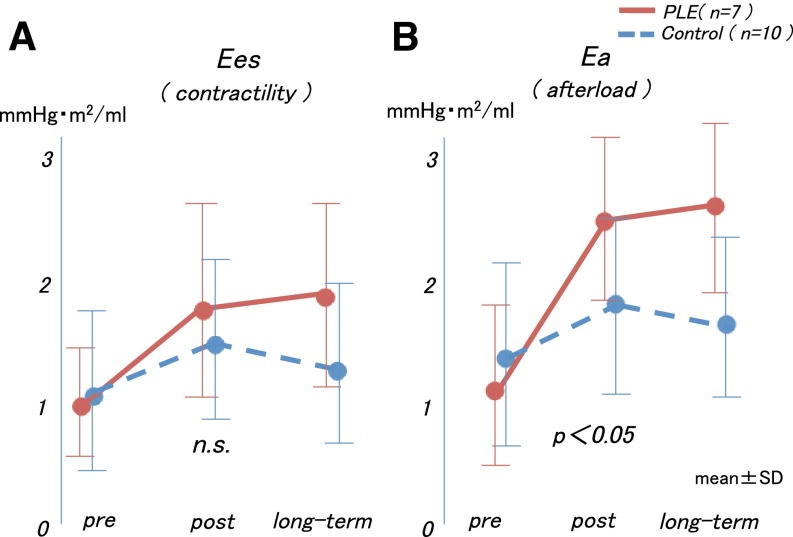
**a** The Ees values did not increase in either group during the long-term postoperative period. **b** The Ea values were not changed in the control group but increased in a time-dependent manner in the PLE group. *Ees* end-systolic elastance, *Ea* arterial elastance, *PLE* protein-losing enteropathy

**Fig. 5:**
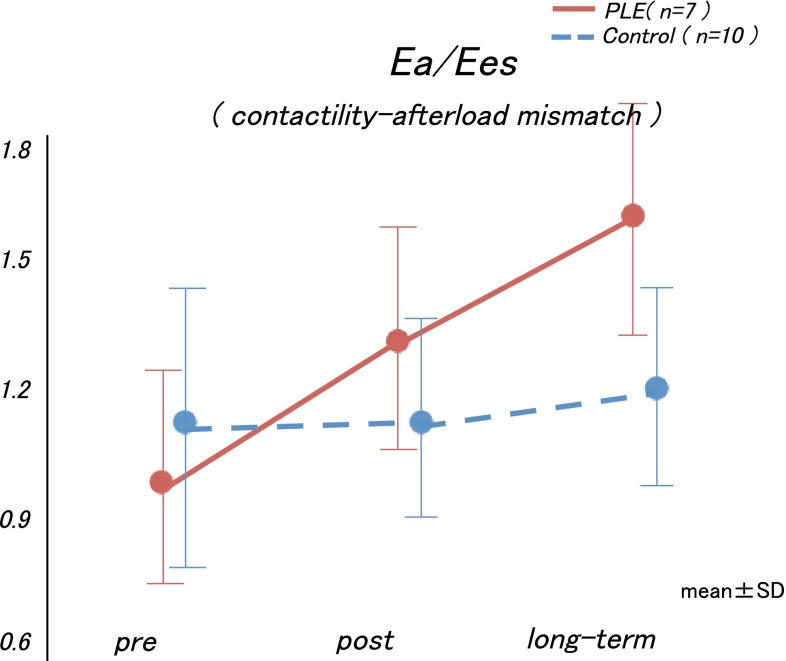
The arterial elastance Ea/Ees values increased significantly and continuously in the PLE group. *Ea* arterial elastance, *Ees* end-systolic elastance, *PLE* protein-losing enteropathy

Furthermore, in the examination of the Ea, Ees, and Ea/Ees values before, after, and during the midterm postoperative period, the Ea value increased after the Fontan operation in both groups, but the subsequent changes in each group were distinctive. Although the Ea values in the control group did not change after the Fontan operation, the Ea values increased in the PLE group after the Fontan operation, yielding a relatively high Ea value during the post-Fontan operation period (interaction effect, *p* = 0.19; group effect, *p* = 0.02; time effect, *p* < 0.0001, Fig. 4a). Meanwhile, the time course in the Ees values between the two groups did not differ significantly during the midterm postoperative period (interaction effect, *p* = 0.93; group effect, *p* = 0.23; Fig. 4b). As a result, the Ea/Ees values increased significantly and continuously in the PLE group (interaction effect, *p* = 0.04; Fig. 5).

**Fig. 6:**
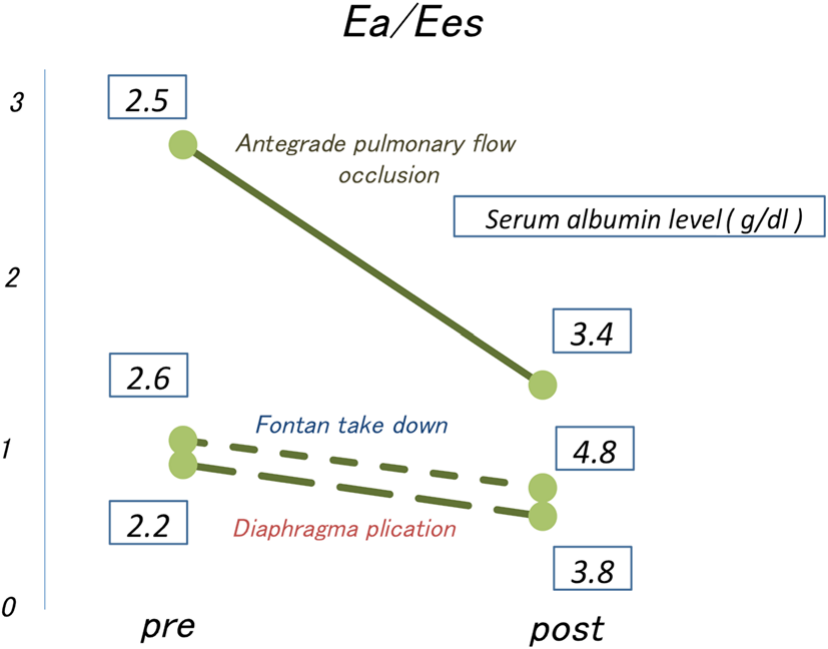
The Ea and Ea/Ees values were decreased after the intervention in all three cases. *Ea* arterial elastance, *Ees* end-systolic elastance

Finally, in all three patients who had undergone surgical intervention to improve Fontan circulation after the onset of PLE, the intervention improved the clinical conditions and serum albumin levels. Moreover, the Ea and Ea/Ees values decreased after the intervention in all three patients (Fig. 6).

## Discussion

In the current study, the Ea and Ea/Ees values increased significantly after the Fontan operation in the PLE group and increased continuously during the postoperative period before the onset of PLE. In addition, we demonstrated that surgical intervention improved the Fontan circulation based on the improvement of the Ea and Ea/Ees values in three patients, even after the onset of PLE, with improvement in the clinical conditions and the serum albumin levels of the patients. To the best of our knowledge, this is the first report to focus on the hemodynamics, especially with regard to contractility–afterload mismatch, in PLE patients.

Several reports have indicated that Fontan circulation led to contractility–afterload mismatch via increased impedance caused by additional connection of the pulmonary vascular bed to the systemic vasculature and deterioration of myocardial contractility. Senzaki et al. [8] reported that the Fontan physiologic mechanism was associated with disadvantageous ventricular power and afterload profiles and had limited ventricular reserve capacity. Therefore, Fontan circulation has profound effects on ventriculoarterial mechanoenergetics in terms of contractility–afterload mismatch and reduced mechanical efficacy.

Meanwhile, in patients who experienced PLE after the Fontan operation, Rychik and Gui-Yang [7] reported significant differences in superior mesenteric artery flow compared with patients who underwent the Fontan operation and did not experience PLE. Furthermore, after the Fontan operation, the vascular resistance of the mesenteric artery in the PLE patients increased compared with that of the patients without PLE [6]. Alterations in mesenteric arterial flow may lay the groundwork for impaired mesenteric perfusion, causing a break in the integrity of the intestinal mucosa and subsequent protein leakage.

We hypothesize that in PLE patients, the systemic low cardiac output of Fontan circulation increases because of contractility–afterload mismatch and that chronic low cardiac output causes redistribution of blood flow away from nonvital organs such as mesenteric organs, leading to impairments of the mesenteric perfusion.

The mechanisms of the afterload increase in the PLE group were not clarified in this study. However, in the current study, the ratios of complications of SVOTS, phrenic nerve palsy, and SSS in the PLE group were significantly higher than in the control group. These complications possibly increase the afterload of the Fontan circulation. Systemic ventricle outflow tract stenosis led to an increase in the afterload of the systemic ventricle and an increase in the contractility–afterload mismatch. Also, phrenic nerve palsy may have led to an increase in the afterload of the systemic ventricle in the cases of Fontan circulation. Achieving a negative pressure in the thoracic cavity of patients with this palsy may be difficult, leading to the increase in systemic venous pressure.

Sick sinus syndrome was demonstrated to induce an increase in mean atrial pressure because of dyssynchrony between the atrium and the ventricle [1]. They described atrial pacing as an alternative treatment for patients who experience PLE after Fontan operation. They suggested that the resolution of clinical and laboratory PLE after atrial pacing likely resulted from augmented cardiac output. Sinus node dysfunction may limit cardiac output through loss of the atrial contribution to ventricular filling. Therefore, we consider that all these complications (SVOTS, phrenic nerve palsy, and SSS) lead to low cardiac output after the increase in the afterload.

In this manuscript, we performed three procedures to improve the Fontan circulation in three patients. Procedures such as takedown of the Glenn circulation, diaphragm plication, and antegrade pulmonary flow occlusion may decrease afterload and improve the contractility–afterload mismatch, thereby improving the low cardiac output. The creation of a communication between the systemic and pulmonary venous chambers as fenestration was reported to be useful in the treatment of PLE in some patients.

Himeshkumar et al. [3] reported that the creation of fenestration by transcatheter improved PLE in patients with Fontan circulation. They reported that the right-to-left shunt and the cardiac index were increased after the procedure. We considered that the creation of the fenestration better improved both the central venous pressure and afterload of the systemic ventricle, as well as the contractility-afterload mismatch and the low cardiac output. We hypothesized that the most important treatment strategy for PLE is to release the vicious circle of low cardiac output in the Fontan circulation. Therefore, aggressive surgical intervention to improve the afterload of the Fontan circulation may improve PLE.

## Study Limitations

This study was retrospective and involved only a small number of patients. Moreover, the study had the limitations of any multicenter clinical study, such as differences in patient evaluation between the centers.

The approximation of Ees and Ea in this study inherently had limitations and did not amount to the measurement obtained with a conductance catheter. Moreover, in this study, we examined the systolic function of the systemic ventricle but not its diastolic function.

We used cardiac catheterization to evaluate the ventricular volume in this study. Magnetic resonance imaging likely is superior to angiography.

## Conclusions

Contractility–afterload mismatch, caused mainly by an increase in the afterload of the systemic ventricle, may play an important role in the development of PLE in patients who undergo the Fontan operation. Therefore, aggressive therapeutic intervention to improve the afterload after the Fontan operation should be recommended to patients with increased afterload and contractility–afterload mismatch, even before the onset of PLE, to prevent the development of PLE.
